# Morphometric Assessment of Pelvic Asymmetry in Domestic Cats and Dogs

**DOI:** 10.3390/ani16050744

**Published:** 2026-02-27

**Authors:** Yusuf Altundağ, Ebru Eravci Yalin, Simge Bayraktar, Murat Karabağlı, Eylem Bektaş Bilgiç, Barış Can Güzel, Alexandra-Andreea Cherșunaru, Aycan Korkmazcan, Nicoleta Manuta, Ozan Gündemir, Mihaela-Claudia Spataru

**Affiliations:** 1 Department of Surgery, Faculty of Veterinary Medicine, Namik Kemal University, Tekirdag 59030, Türkiye; yaltundag@nku.edu.tr; 2 Department of Surgery, Faculty of Veterinary Medicine, Istanbul University-Cerrahpasa, Istanbul 34320, Türkiye; ebru.eravciyalin@iuc.edu.tr (E.E.Y.); simge.ugur@iuc.edu.tr (S.B.); murat.karabagli@iuc.edu.tr (M.K.); eylem.bilgic@iuc.edu.tr (E.B.B.); 3 Department of Anatomy, Faculty of Veterinary Medicine, Siirt University, Siirt 56100, Türkiye; baris.guzel@siirt.edu.tr; 4 Faculty of Veterinary Medicine, Ion Ionescu de la Brad Iasi University of Life Science, 700489 Iasi, Romania; alexandra.chersunaru@iuls.ro (A.-A.C.); mihaela.spataru@iuls.ro (M.-C.S.); 5 Institute of Graduate Studies, Istanbul University-Cerrahpasa, Istanbul 34320, Türkiye; aycan.korkmazcan@ogr.iuc.edu.tr (A.K.); nicoletamanuta@ogr.iuc.edu.tr (N.M.); 6 Department of Anatomy, Faculty of Veterinary Medicine, Istanbul University-Cerrahpasa, Istanbul 34320, Türkiye; 7 Osteoarchaeology Practice and Research Centre, Istanbul University-Cerrahpasa, Istanbul 34320, Türkiye

**Keywords:** 3D geometric morphometrics, directional asymmetry, fluctuating asymmetry, object symmetry, sex differences, measurement error, veterinary anatomy

## Abstract

This study used 3D landmark-based geometric morphometrics under an object-symmetry framework to quantify pelvic asymmetry in domestic cats and dogs while explicitly accounting for measurement error through replicate digitizations. Procrustes ANOVA revealed significant components of both directional asymmetry (DA) and fluctuating asymmetry (FA) in the overall dataset, and DA was further visualized as a structured, non-random pattern across the landmark configuration. Measurement error remained smaller than the FA component, yielding high repeatability, indicating that the detected asymmetry patterns were not driven by landmarking imprecision. Group-wise summaries are presented as descriptive patterns of the sample (rather than direct between-group inference). In these descriptive summaries, cats tended to show a more coherent DA pattern, whereas dogs showed greater individual variation consistent with a relatively stronger FA component; males also tended to exhibit greater FA-related dispersion than females. In regression models of FA, age showed no association, body mass exhibited only a weak trend, and sex emerged as a significant predictor, while species showed no detectable effect when covariates were included. Collectively, these results support the hypothesis that pelvic shape contains both systematic (DA) and individual-specific (FA) asymmetry components, with sex-related differences in FA magnitude, but limited evidence for age- or weight-related effects within the sampled range. The study provides a repeatable framework and baseline reference for pelvic asymmetry in cats and dogs.

## Simple Summary

Pelvic bones are expected to exhibit left–right symmetry; however, small differences between sides can arise due to growth, daily movement, and individual life history. Evidence was found for two patterns: a small but consistent left–right difference across the overall sample and additional individual-to-individual variation in asymmetry. High consistency was observed across repeated landmark digitizations, indicating that the detected patterns were not primarily driven by digitizing error. When groups were compared, the patterns described for cats and dogs reflect descriptive summaries of the sample rather than formal between-group statistical inference. In these descriptive summaries, cats tended to exhibit a stronger and more consistent left–right pattern, whereas dogs showed greater individual variation. Differences in asymmetry magnitude were also observed between males and females. No clear association was detected with age, and only a weak trend was observed with body mass. These findings provide a reliable reference for understanding pelvic shape differences in cats and dogs and may be useful for veterinary anatomy and comparative research.

## 1. Introduction

The pelvis forms the bony foundation of the hindlimb and the pelvic canal, integrating locomotor function with reproductive and visceral support in domestic mammals [1,2]. In cats and dogs, pelvic morphology is expected to vary with overall body size, sex-related differences, and breed/class-associated conformation, making it a useful structure for comparative and applied veterinary morphology [3,4]. Recent geometric morphometric (GM) studies have demonstrated measurable pelvic shape variation in crossbreed cats and among different dog classes, supporting the use of landmark-based shape analysis to quantify pelvic form differences in these species [3,4]. More broadly, pelvic shape variation is well recognized as functionally informative in mammals (e.g., linked to obstetric and locomotor constraints), reinforcing the value of pelvic GM in comparative contexts [5,6,7].

Although the pelvis is bilaterally organized, perfect left–right symmetry is rarely achieved in biological structures, and small deviations can arise through growth processes, mechanical loading, and individual life-history effects [8,9,10]. Two major asymmetry components are typically distinguished: directional asymmetry (DA), representing a consistent left–right bias at the population level, and fluctuating asymmetry (FA), representing non-directional, individual-specific deviations around symmetry that are often discussed in the context of developmental instability [8,9,10]. Because these components may occur simultaneously in skeletal traits, separating DA from FA is important for interpreting whether asymmetry reflects systematic patterning versus mainly individual-level variation [10,11,12].

Geometric morphometrics provides a powerful framework for describing and comparing shape quantitatively using homologous anatomical landmarks [13,14,15]. Within GM, bilateral symmetry can be examined in detail using object symmetry approaches in which reflected (mirrored) configurations are incorporated into the analysis; this allows shape variation to be partitioned into among-individual differences, directional asymmetry (DA), fluctuating asymmetry (FA), and, when replicate measurements are available, measurement error [11,12]. Such partitioning enables biological asymmetry components to be evaluated independently of technical error and supports reliable comparisons of asymmetry patterns among groups such as species and sexes [11,12]. Accordingly, the present study aimed to quantify DA and FA components in the pelvis of cats and dogs and to assess their relationships with basic biological variables.

The pelvis is a bilaterally symmetric structure, yet subtle left–right differences can arise from developmental and functional sources, potentially producing both directional asymmetry and fluctuating asymmetry [16,17]. Despite the widespread use of geometric morphometrics for quantifying asymmetry, pelvic asymmetry patterns in domestic carnivores and their potential association with biological factors remain insufficiently characterized. Previous geometric morphometric studies in domestic carnivores have primarily focused on overall pelvic shape variation (e.g., across cat populations and among dog classes) rather than explicitly quantifying pelvic asymmetry and its components [3,4]. In particular, comparative interspecific assessments that partition directional asymmetry and fluctuating asymmetry while simultaneously estimating measurement error from replicate digitizations remain scarce. This gap limits the interpretability and comparability of subtle asymmetry signals in clinical CT-derived datasets. Here, we address this by applying an object-symmetry framework with replicate-based error partitioning to provide a repeatable baseline reference for pelvic asymmetry in domestic cats and dogs.

We hypothesized (H1) that pelvic shape would show both a systematic directional component, consistent with shared functional loading and habitual locomotor/postural biases, and an individual-specific component, reflecting developmental and/or life-history-related variation. (H2) We predicted interspecific differences in the relative prominence of DA versus FA, expecting dogs to exhibit comparatively higher individual-to-individual asymmetry variance given their broader size range and heterogeneity associated with breed-driven selection, whereas cats may show a more coherent directional pattern. (H3) We predicted sex differences in FA magnitude, consistent with sex-linked differences in body size, pelvic functional demands, and growth-related developmental trajectories.

## 2. Materials and Methods

### 2.1. Samples

A retrospective dataset of whole-body computed tomography (CT) examinations acquired for routine clinical purposes between 2022 and 2026 was used. Only cases without any apparent abnormality affecting the pelvis or the skeletal system were included. Prior to the study, all CT images were screened by an expert radiologist, and individuals showing pelvic fractures, malformations, degenerative/orthopedic pathology, implants, or any other condition that could influence pelvic morphology were excluded. To avoid potential confounding effects of incomplete skeletal maturation, animals younger than 1 year were not included.

For each animal, clinical records were used to obtain species, breed, sex, age (years), and body mass (kg). The final dataset comprised 99 individuals with complete metadata and replicate landmark sets, including 41 cats and 58 dogs. Overall, ages ranged from 1 to 14 years (mean ± SD: 6.94 ± 4.48), and body mass ranged from 2 to 57 kg (mean ± SD: 14.35 ± 14.32). The sex distribution consisted of 46 females and 53 males (cats: 21 F/20 M; dogs: 25 F/33 M).

Because the dataset was retrospective and derived from routine clinical CT examinations, the sample represents a convenience population rather than a random cross-section of the general cat and dog population. Consequently, the findings should be interpreted primarily as a baseline reference for clinically imaged adult individuals without apparent pelvic pathology, rather than as fully population-representative estimates.

### 2.2. CT Acquisition and 3D Modeling

All examinations were performed using a 128-slice CT scanner (SOMATOM Definition AS+; Siemens Healthineers, Forchheim, Germany) under a single standardized acquisition protocol. Image data were obtained with a 0.6 mm slice thickness, a tube voltage of 120 kVp, and a tube current of 240 mAs.

Three-dimensional reconstruction of the pelvic region was conducted in 3D Slicer (version 5.2.2) using the original DICOM datasets [18]. The pelvis was segmented from surrounding tissues and artifacts were minimized through careful refinement of the segmentation masks to obtain clean bony structures. Following segmentation, surface meshes were generated from the final masks and exported as polygonal models in PLY format for subsequent landmark-based analyses.

### 2.3. Landmarking

**Figure 1:**
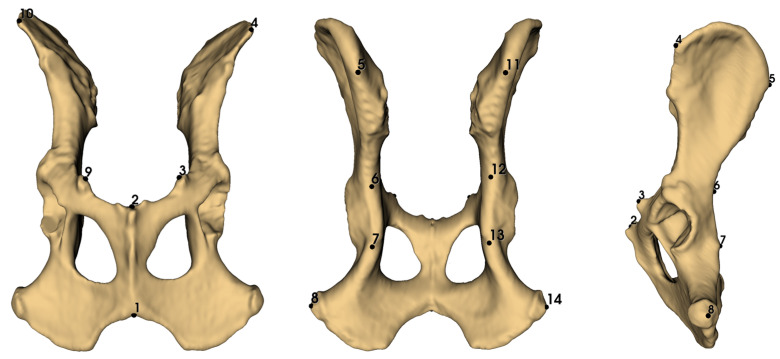
Three-dimensional pelvic surface model illustrating the 14-landmark configuration used for geometric morphometrics and object-symmetry analyses (example specimen: adult male Labrador Retriever): (1) Most caudal point of the pelvic symphysis at the midline; (2) Most cranial point of the pelvic symphysis at the midline (pubic symphyseal region); (3–9) Most prominent point of the iliopubic (iliopectineal) eminence on the cranial border of the acetabulum (right/left); (4–10) Most cranial point of the cranial iliac spine on the ilium (right/left); (5–11) Most caudal point of the dorsal iliac spine adjacent to the sacroiliac region (right/left); (6–12) Deepest point of the greater sciatic notch (*incisura ischiadica major*) (right/left); (7–13) Most prominent point of the ischial spine (right/left); (8–14) Most caudolateral point of the ischial tuberosity (right/left).

Landmark digitization was performed in 3D Slicer (version 5.2.2) on the exported pelvic surface meshes [18,19,20]. To ensure consistency, all landmarking was carried out by a single observer using a standardized landmark protocol. Because landmarking was performed by a single investigator, repeatability estimates reflect intra-observer consistency; inter-observer reliability was not assessed in the present study. A total of 14 three-dimensional landmarks were recorded for each specimen (Figure 1). The landmark configuration was selected to provide a robust and repeatable representation of overall pelvic geometry in clinically derived CT data. Landmarks were restricted to clearly identifiable, anatomically homologous points that could be located consistently across individuals and across species, minimizing ambiguity due to variable image quality or subtle anatomical variation. The chosen set spans the major pelvic regions (ilium, ischium, pubis and the acetabular area), thereby capturing both global shape variation and left–right differences relevant to object symmetry analyses.

Landmarks 1 and 2 were placed on midline anatomical locations, whereas the remaining landmarks were positioned as bilateral pairs on the left and right sides of the pelvis, enabling object-symmetry analyses. All landmarks were defined at clearly identifiable anatomical points to maximize repeatability across specimens and between replicate digitizations.

### 2.4. Assessment of Measurement Error and Landmark Repeatability

To quantify intra-observer measurement error and to ensure that asymmetry signals were not driven by digitizing imprecision, all specimens were landmarked twice by the same observer in two separate digitization sessions. These repeated landmark configurations were treated as replicate measurements and were incorporated into the symmetry framework as an additional factor. Under an object-symmetry Procrustes ANOVA design, replicate information allows the total shape variation to be partitioned into biological components (including DA and FA) while explicitly estimating measurement error from the replicate term and its interactions. This approach is widely recommended in geometric morphometric studies because landmarking error can otherwise inflate or obscure biological effects, particularly when asymmetry components are subtle [12,21]. Repeatability of landmark placement was evaluated by comparing the variance attributable to asymmetry with the variance attributable to replicate digitization, following standard practices in the geometric morphometrics literature [12,21].

### 2.5. Statistical Analyses

All statistical analyses were conducted in R (version 4.4.2) [22]. Landmark coordinates were organized as three-dimensional landmark configurations (14 landmarks × 3 coordinates) for each specimen, and bilateral landmark correspondence was defined for object-symmetry analyses. Landmarks 1–2 were treated as midline landmarks, and the remaining landmarks were defined as paired bilateral landmarks with the following left–right matches: 3–9, 4–10, 5–11, 6–12, 7–13, and 8–14.

Bilateral symmetry decomposition was performed under an object symmetry framework using functions implemented in geomorph (v. 4.0.9) [23]. Procrustes ANOVA based on randomized residual permutation procedures was used to partition shape variation into the population-level side effect (directional asymmetry, DA), the individual × side interaction (fluctuating asymmetry, FA), and, because replicate digitizations were available, the individual × side × replicate term (measurement error, ME) [11,12]. Statistical significance was evaluated using 1000 permutations, and sequential (Type I) sums of squares were used for hypothesis testing. We used Type I (sequential) sums of squares because the model terms follow a biologically and analytically motivated order (species and sex effects, followed by size- and age-related covariates, and then the nested breed structure), allowing interpretable partitioning of variance in a stepwise manner. This choice is consistent with our primary aim of evaluating how major grouping factors and key covariates contribute to asymmetry variation in a structured, hierarchical design.

To summarize digitizing precision, landmark repeatability was quantified by comparing the mean square for FA with the mean square for ME, and a repeatability coefficient was calculated as [21]:

Repeatability = MS(FA)/[MS(FA) + MS(ME)]

In addition to the overall analysis, symmetry decomposition was repeated for predefined subsets (species, sex, and species × sex) to obtain group-wise DA/FA summaries.

To account for dog breed heterogeneity, we explicitly modeled breed structure in the asymmetry analyses. We fitted a model including breed nested within species (Species: Breed) alongside Species, Sex, Species × Sex, log (Weight), and Age. In addition, we performed a dog-only model including Breed as a fixed factor (with Sex, log (Weight), and Age as covariates) to evaluate breed effects within dogs. We did not apply a separate ‘allometry-correction’ step based on centroid size (e.g., regressing shape on size and analyzing residuals). Instead, size-related effects were addressed by including body mass (log-transformed) as a covariate in the multivariable models evaluating asymmetry shape and unsigned FA, treating weight as a practical proxy for overall body size in this retrospective clinical dataset.

For evaluating covariate effects on asymmetry magnitude, the unsigned FA index (individual-level FA magnitude) obtained from the symmetry decomposition was analyzed using permutation-based linear modeling. Models were fitted to assess associations between unsigned FA and age and body mass, with an extended model including species and sex as additional predictors; significance was assessed by residual randomization with 1000 permutations [24].

## 3. Results

Procrustes ANOVA (RRPP, 1000 permutations) performed under an object symmetry framework for the pelvic 3D landmark dataset showed that directional asymmetry and fluctuating asymmetry were significant (Table 1). Notably, while the DA term was statistically detectable, it represented a relatively small fraction of the total variance, indicating that the directional component is subtle in magnitude compared with other sources of variation.

Measurement error was assessed using the ind:side:replicate term (Df = 198, SS = 0.0268, MS = 0.000135, Rsq = 0.00332). The mean square for measurement error (MS = 0.000135) was lower than the mean square for FA (MS = 0.002219). Accordingly, landmark repeatability was high, and the repeatability coefficient calculated from the replicate measurements was approximately 0.94.

**Figure 2:**
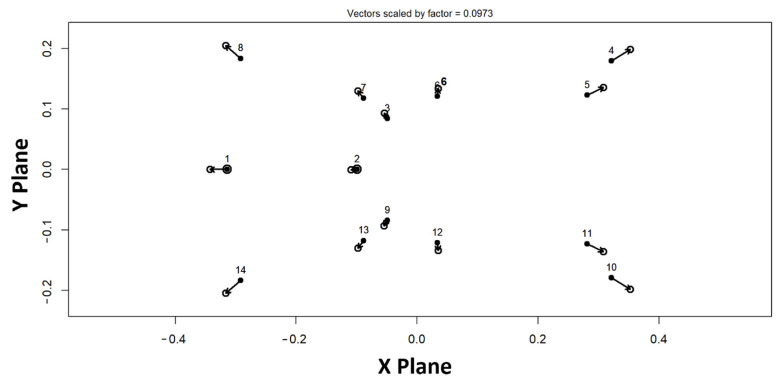
Projected 2D vector plot (XY plane) visualizing directional asymmetry (DA) in pelvic shape. Vectors indicate the direction and relative magnitude of systematic left–right deviations at each landmark, plotted as displacements from the symmetric mean configuration.

Directional asymmetry was further visualized using a projected 2D vector plot of the DA component in the XY plane (Figure 2). In this plot, vectors represent the direction and relative magnitude of systematic left–right shape differences at each landmark, shown as displacements from the symmetric mean configuration. Consistent with the significant DA term in the Procrustes ANOVA, the DA vectors indicate a structured, non-random pattern of directional deviation across the landmark configuration (Figure 2).

**Figure 3:**
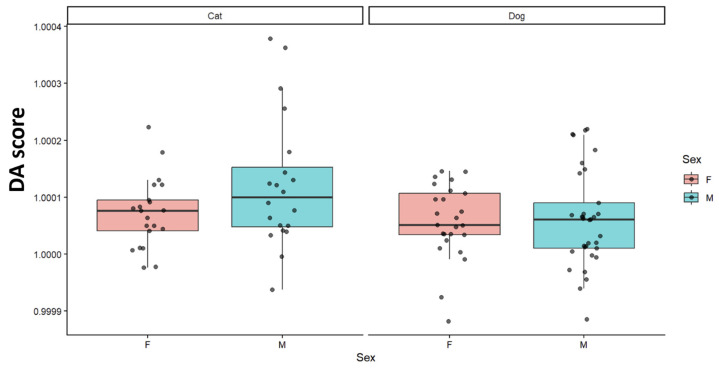
Distribution of signed DA scores by sex within each species. Boxplots summarize central tendency and dispersion, and points represent individual observations. Signed DA scores represent the individual-level projection along the directional asymmetry axis derived from the symmetry decomposition; group-wise patterns are shown as descriptive summaries. F: Female, M: Male. 46 females and 53 males (cats: 21 F/20 M; dogs: 25 F/33 M).

Signed directional asymmetry scores were summarized by sex within each species to visualize the distribution of DA at the individual level (Figure 3). The boxplots and individual data points show the spread and central tendency of signed DA scores for females and males in cats and dogs. Across groups, the distributions partially overlap, while differences in the location and dispersion of the signed DA scores between sexes can be assessed visually within each species (Figure 3).

According to Table 2, the pelvic 3D landmark dataset exhibits both directional asymmetry and fluctuating asymmetry at the overall sample level. For the subset analyses (species, sex, and species × sex), the reported tests evaluate the presence of DA and FA within each subset. Descriptive comparisons of component prominence across subsets are therefore presented as observed patterns rather than formal between-group inference. In these descriptive summaries, cats appeared to show a more coherent DA pattern, whereas dogs showed greater individual variation consistent with a stronger FA-related dispersion. With respect to sex, males appeared to show greater FA-related dispersion than females. When the data were partitioned by species × sex, both components remained evident in the cat subgroups, whereas in the dog subgroups FA remained prominent while the directional signal appeared weaker in the descriptive summaries.

Regarding measurement error, MS(FA) is consistently higher than MS(ME) across all groups, indicating that the variation attributable to asymmetry exceeds the variation introduced by repeated landmarking. This pattern supports low measurement error and high consistency between replicate digitizations. In line with this, the repeatability values reported in Table 2 are generally high, further indicating that the results are robust and reliable, and not driven by landmarking imprecision. These subset analyses evaluate asymmetry within each group; any cross-group contrasts mentioned are descriptive summaries and are not presented as direct between-group inference.

When breed structure was incorporated into the asymmetry model, the nested breed term (Species: Breed) explained a large fraction of variation and was significant (Df = 36, F = 5.99, R^2^ = 0.461, *p* = 0.001), indicating that breed-related heterogeneity contributes substantially to variance in pelvic asymmetry shape. In the dog-only analysis, Breed remained strongly significant (Df = 29, F = 6.67, R^2^ = 0.828, *p* = 0.001), while log (Weight) also showed a significant effect (F = 4.07, *p* = 0.002); Age was not significant (*p* = 0.702).

**Figure 4:**
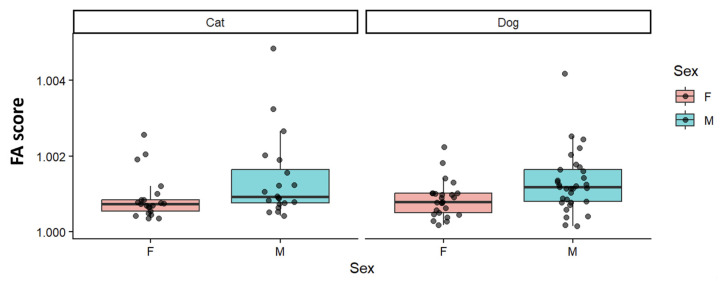
FA by sex within cats and dogs. FA represents individual-level asymmetry magnitude derived from the object-symmetry decomposition; group-wise plots summarize within-group variation. F: Female, M: Male. 46 females and 53 males (cats: 21 F/20 M; dogs: 25 F/33 M).

Table 3 presents the RRPP-based regression results evaluating the association between the unsigned FA index and the predictors included in the model. Unsigned FA showed no association with age and no detectable effect of species (Table 3). Body mass displayed a weak/marginal trend with unsigned FA, but this effect did not reach statistical significance (Table 3). In contrast, sex was significantly associated with unsigned FA, indicating differences in FA magnitude between females and males (Figure 4).

**Figure 5:**
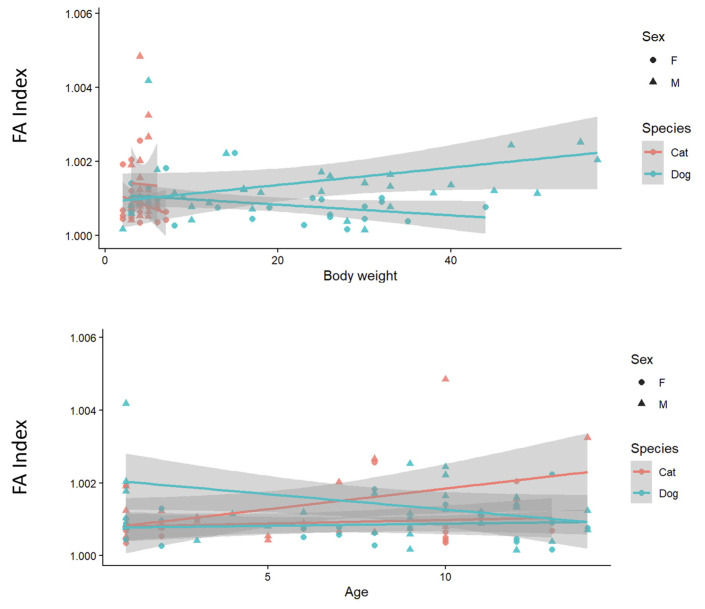
Relationships between pelvic unsigned FA and body mass (kg) and age (years) in cats and dogs (F: Female, M: Male).

Body mass and age were further explored visually in relation to unsigned FA (Figure 5). Across the sampled range, FA values showed only limited variation with both predictors, and the fitted trends were generally weak. Any apparent patterns differed slightly between cats and dogs, but overall changes in FA across body mass and age were modest (Figure 5).

## 4. Discussion

Overall, this study aimed to quantify pelvic asymmetry in domestic cats and dogs using 3D landmark-based geometric morphometrics under an object-symmetry framework, while explicitly separating biological asymmetry from digitizing error through replicate landmarking. The results support this objective: both directional asymmetry and fluctuating asymmetry were detected at the whole-sample level, and the measurement error term remained clearly smaller than the FA component, yielding high repeatability. Together, these findings indicate that the observed asymmetry patterns are not an artefact of landmarking imprecision, and that the analytical pipeline provides a reliable basis for comparing asymmetry components across groups.

The first hypothesis predicting measurable contributions of both DA and FA, was supported. The significant DA term, together with the DA vector visualization, indicates that left–right differences are not purely random but include a detectable directional component across the landmark configuration. However, the variance partitioning suggests that this directional component is relatively modest in magnitude and should therefore be interpreted as a subtle baseline bias rather than a dominant driver of pelvic shape variation. Such directional signals may be consistent with systematic influences that act similarly across individuals (for example, shared functional demands, habitual postures, or common locomotor patterns). However, the present study does not include direct biomechanical or kinematic measurements, and therefore these explanations should be regarded as plausible interpretations rather than demonstrated causes. Likewise, the FA component is often discussed in relation to developmental instability and individual-specific life-history or mechanical factors, but distinguishing among these candidate mechanisms would require targeted designs (e.g., longitudinal sampling, controlled activity histories, or biomechanical data).

The second hypothesis suggested that asymmetry patterns might differ between species due to differences in pelvic biomechanics and developmental trajectories. In our dataset, however, the expected species-level contrasts in asymmetry were not as distinct as anticipated. A plausible explanation is that dogs represent a morphologically heterogeneous group, and that this within-dog diversity can increase variance in asymmetry measures, thereby making broader species-level differences harder to resolve in a clinical sample. To address this issue directly, we incorporated breed structure into the analytical design by explicitly modeling breed effects within species, and we also evaluated the dog sample separately while accounting for body-size differences. These additional analyses supported the interpretation that breed-related heterogeneity is a major contributor to asymmetry variation in dogs. Accordingly, interspecific comparisons of FA-related dispersion should be interpreted in the context of breed diversity, and future work would benefit from breed- or size-stratified sampling designs to disentangle species effects from within-species morphological diversity.

The third hypothesis, predicting associations of FA with age or body mass, was only weakly supported. Age showed no association with FA, while body mass displayed only a marginal trend that did not reach conventional significance. This outcome may indicate that FA in pelvic shape is relatively stable across the sampled age range, or that cross-sectional age and body mass are not sufficiently sensitive proxies for the factors that shape pelvic asymmetry. It is also possible that the available ranges of age and weight, breed-level heterogeneity, and unmodeled sources of variation (e.g., neuter status, activity level, reproductive history, or subtle differences in specimen orientation/positioning before landmarking) limited the detection of stronger effects. Future work with larger, more balanced samples and standardized positioning protocols and, where possible, breed-specific or allometry-aware models, would help clarify how biomechanics and life-history factors influence the partitioning of pelvic asymmetry into DA and FA components.

Asymmetry patterns in the pelvis can vary among species, potentially reflecting differences in developmental stability, population structure, and scaling effects. Prior work has highlighted that asymmetry estimates can be sensitive to size (allometry) and that cross-group interpretations can be misleading if scaling and sampling structure are not considered [25,26]. In the present study, the within-group symmetry framework establishes that both DA and FA are present in the dataset, whereas any apparent differences in the relative prominence of components across cats and dogs should be interpreted as descriptive patterns of this clinical sample [27]. Consistent with this, dogs represent a morphologically diverse population shaped by strong human-directed selection, and breed-related heterogeneity can contribute substantially to among-individual dispersion in asymmetry measures [28,29,30]. In cats, comparatively constrained diversity patterns may yield a more coherent directional pattern in descriptive summaries. Future work based on structured sampling (e.g., breed- or size-stratified designs) will be especially useful for disentangling species effects from within-species morphological diversity [31]. Because this retrospective design does not include direct biomechanical or kinematic measures (e.g., gait metrics or loading estimates), any functional interpretations should be viewed as hypotheses for future testing rather than demonstrated mechanisms.

For the asymmetry findings obtained in this study to be considered reliable, low measurement error and high repeatability are critical. Indeed, the geometric morphometrics literature has shown that variation attributable to biological asymmetry should clearly exceed variation introduced by digitizing error. For example, one study reported, based on Procrustes ANOVA, that among-individual shape differences were higher than measurement error [32]. Another study found that individual variation was 7–17 times greater than total measurement error, with error accounting for only 4–12% of the total variance [33]. In this context, observing MS(FA) > MS(ME) in our dataset is expected, and the agreement between repeated digitizations was high. Overall, the high repeatability values confirm that landmark placement was consistent and that the asymmetry results were not driven by measurement error.

Evidence for a pronounced effect of age and body mass on pelvic asymmetry is limited. In particular, once growth has been completed, an effect of age on asymmetry is generally not observed. For example, one study in cats reported that age or body mass did not have a significant effect on pelvic size [3]. Similarly, it has been shown that the level of directional asymmetry can be independent of body size, with only a very weak size-related association with shape [34]. In our findings, the “marginal” effect of body mass likely reflects a very small allometric influence on pelvic shape. Overall, in adult individuals, neither increasing age nor reasonable differences in body mass appear to substantially alter pelvic asymmetry.

Establishing a baseline description of pelvic asymmetry in clinically imaged cats and dogs has practical value for both applied veterinary contexts and future comparative work. Clinically, a reference baseline helps distinguish subtle, expected left–right differences from asymmetry patterns that may warrant closer scrutiny in CT-based assessments, particularly when overt pelvic pathology has been excluded. This is relevant for interpretation of pelvic morphology in routine imaging and may support decision-making in contexts where symmetry assumptions implicitly influence evaluation, such as follow-up of orthopedic conditions, post-traumatic monitoring, and pre-operative planning that relies on contralateral comparison. In addition, documenting that asymmetry components can be detected even in apparently normal pelves encourages cautious interpretation of “minor” side differences and supports the use of replicate-aware workflows when asymmetry is of interest. From a broader perspective, the baseline provided here offers a standardized starting point for comparative morphology studies that aim to evaluate how sampling structure (e.g., breed diversity), sex-related factors, or life-history variables modulate asymmetry patterns across populations. Importantly, while several candidate mechanisms (e.g., functional or developmental influences) can be discussed as plausible interpretations, testing such explanations would require targeted designs that integrate structured sampling and direct functional or longitudinal data.

A key limitation of this study is that, although detailed metadata were available for most individuals, reproductive history in females (pregnancy) was not recorded. This is potentially important because pregnancy and parturition can alter pelvic biomechanics through hormone-mediated ligament laxity and remodeling of pelvic and adjacent musculoskeletal tissues, and repeated pregnancies may contribute to longer-term changes in loading patterns and shape variance. Consequently, unmeasured differences in parity among females could act as an additional source of within-sex variability and may partly mask or modify associations between FA and other predictors such as body mass or age. Future studies would benefit from including reproductive status indicators (nulliparous vs. multiparous, number of litters, time since last pregnancy) to evaluate whether reproductive history contributes to pelvic FA and to better isolate sex and size related effects. In addition, because the dataset is retrospective and clinically derived, the sample reflects a convenience population rather than a random representation of the broader cat and dog populations. Although individuals with apparent pelvic pathology were excluded, unmeasured clinical and demographic factors may still influence sample composition, which may limit population-level generalizability of effect magnitudes. A further limitation is that measurement error was quantified only via intra-observer replicates. Although this approach supports consistent DA/FA partitioning within a single digitizer, it does not capture variability introduced by different observers.

## 5. Conclusions

This study provides a replicate-aware, 3D geometric morphometric assessment of pelvic asymmetry in domestic cats and dogs under an object-symmetry framework. The results demonstrate that pelvic shape includes both a significant directional asymmetry component, reflecting a subtle but structured population-level left–right bias, and a significant fluctuating asymmetry component, reflecting individual-specific variation. Importantly, measurement error estimated from replicate landmark digitizations remained lower than the FA component across the overall dataset and group-wise analyses, supporting high repeatability and indicating that the detected asymmetry patterns were not driven by landmarking imprecision.

## Figures and Tables

**Table 1:** Procrustes ANOVA summarizing whole-sample partitioning of pelvic shape asymmetry into directional asymmetry (DA; population-level side effect) and fluctuating asymmetry (FA; individual × side interaction).

Asymmetry Component	Df	SS	MS	R^2^	F	Z	*p*
DA	1	0.0148	0.014844	0.00184	6.6897	4.0752	0.001
FA	98	0.2175	0.002219	0.02700	16.4077	26.9125	0.001

**Table 2:** Group-wise Procrustes ANOVA summaries for directional asymmetry (DA), fluctuating asymmetry (FA), and measurement error (ME) derived from replicate digitizations under an object-symmetry framework.

Group	N	DA (F)	DA (*p*)	FA (F)	FA (*p*)	MS (FA)	MS (ME)	Repeatability
All	99	6.69	0.001	16.4	0.001	0.00222	0.000135	0.939
Cat	41	6.16	0.001	7.97	0.001	0.00203	0.000255	0.875
Dog	58	2.99	0.009	44.0	0.001	0.00232	0.0000528	0.977
Female	46	3.49	0.001	13.1	0.001	0.00166	0.000126	0.924
Male	53	3.99	0.002	18.9	0.001	0.00271	0.000143	0.947
Cat-Female	21	2.80	0.008	6.48	0.001	0.00166	0.000256	0.846
Cat-Male	20	4.06	0.009	9.58	0.001	0.00242	0.000253	0.896
Dog-Female	25	1.93	0.086	87.8	0.001	0.00166	0.0000189	0.989
Dog-Male	33	1.64	0.140	36.5	0.001	0.00286	0.0000785	0.973

DA corresponds to the Side effect (systematic left–right difference within each group). FA corresponds to the Individual × Side interaction (individual-specific asymmetry within each group). Reported F and *p* values test the presence of DA/FA within each subset, not differences between subsets. Repeatability was calculated as MS(FA)/[MS(FA) + MS(ME)]. MS: Mean Square, ME: Measurement Error.

**Table 3:** ANOVA results for the effects of age, body mass, species, and sex on FA.

Effect	R^2^	F	Z	*p*
Age	5.45 × 10^−5^	0.005	−1.678	0.952
Weight	0.031	3.094	1.376	0.077
Species	0.006	0.671	0.264	0.425
Sex	0.067	7.037	2.276	0.009

R^2^ = proportion of total shape variation explained by the effect (effect size); F = F-statistic; Z = effect size expressed as a standardized score in the permutation framework; *p* = permutation-based probability value (*p*-value).

## Data Availability

The data presented in this study are available upon request from the corresponding author (O.G.).

## References

[1] Singh B. (2018). Dyce, Sack, and Wensing’s Textbook of Veterinary Anatomy.

[2] Hermanson J.W., De Lahunta A. (2018). Miller and Evans’ Anatomy of the Dog-E-Book.

[3] Manuta N., Gündemir O., Yalin E.E., Karabağli M., Uçmak Z.G., Dal G.E., Gürbüz İ. (2023). Pelvis shape analysis with geometric morphometry in crossbreed cats. Anat. Histol. Embryol..

[4] Özkan E., Avanus K., Manuta N., Aydoğdu S., Altundağ Y. (2024). Shape variations of pelvis in different classes of dogs using geometric morphometry. Anat. Histol. Embryol..

[5] Gonzalez P.N., Bernal V., Perez S.I. (2009). Geometric morphometric approach to sex estimation of human pelvis. Forensic Sci. Int..

[6] Kurki H.K., Decrausaz S.L. (2016). Shape variation in the human pelvis and limb skeleton: Implications for obstetric adaptation. Am. J. Phys. Anthropol..

[7] Betti L., von Cramon-Taubadel N., Manica A., Lycett S.J. (2013). Global geometric morphometric analyses of the human pelvis reveal substantial neutral population history effects, even across sexes. PLoS ONE.

[8] Palmer A.R., Strobeck C. (1986). Fluctuating asymmetry: Measurement, analysis, patterns. Annu. Rev. Ecol. Syst..

[9] Parsons P.A. (1992). Fluctuating asymmetry: A biological monitor of environmental and genomic stress. Heredity.

[10] Graham J.H., Raz S., Hel-Or H., Nevo E. (2010). Fluctuating asymmetry: Methods, theory, and applications. Symmetry.

[11] Klingenberg C.P., McIntyre G.S. (1998). Geometric morphometrics of developmental instability: Analyzing patterns of fluctuating asymmetry with Procrustes methods. Evolution.

[12] Klingenberg C.P. (2015). Analyzing fluctuating asymmetry with geometric morphometrics: Concepts, methods, and applications. Symmetry.

[13] Mitteroecker P., Gunz P. (2009). Advances in geometric morphometrics. Evol. Biol..

[14] Bookstein F.L. (1991). Morphometric Tools for Landmark Data: Geometry and Biology.

[15] Zelditch M.L., Swiderski D.L., Sheets H.D. (2012). Geometric Morphometrics for Biologists: A Primer.

[16] Boulay C., Tardieu C., Bénaim C., Hecquet J., Marty C., Prat-Pradal D., Legaye J., Duval-Beaupère G., Pélissier J. (2006). Three-dimensional study of pelvic asymmetry on anatomical specimens and its clinical perspectives. J. Anat..

[17] Tobolsky V.A., Kurki H.K., Stock J.T. (2016). Patterns of directional asymmetry in the pelvis and pelvic canal. Am. J. Hum. Biol..

[18] Pieper S., Halle M., Kikinis R. (2004). 3D Slicer. Proceedings of the 2nd IEEE International Symposium on Biomedical Imaging: Nano to Macro.

[19] Rolfe S., Pieper S., Porto A., Diamond K., Winchester J., Shan S., Kirveslahti H., Boyer D., Summers A., Maga A.M. (2021). SlicerMorph: An open and extensible platform to retrieve, visualize and analyse 3D morphology. Methods Ecol. Evol..

[20] Gündemir O., Szara T. (2025). Morphological patterns of the European bison (Bison bonasus) skull. Sci. Rep..

[21] Fruciano C. (2016). Measurement error in geometric morphometrics. Dev. Genes Evol..

[22] R Core Team (2016). R: A Language and Environment for Statistical Computing.

[23] Baken E.K., Collyer M.L., Kaliontzopoulou A., Adams D.C. (2021). geomorph v4. 0 and gmShiny: Enhanced analytics and a new graphical interface for a comprehensive morphometric experience. Methods Ecol. Evol..

[24] Collyer M.L., Adams D.C. (2018). RRPP: An r package for fitting linear models to high-dimensional data using residual randomization. Methods Ecol. Evol..

[25] Leung B. (1998). Correcting for allometry in studies of fluctuating asymmetry and quality within samples. Proc. R. Soc. London. Ser. B Biol. Sci..

[26] Windig, Nylin (1999). How to compare fluctuating asymmetry of different traits. J. Evol. Biol..

[27] Graham J.H., Emlen J.M., Freeman D.C., Leamy L.J., Kieser J.A. (1998). Directional asymmetry and the measurement of developmental instability. Biol. J. Linn. Soc..

[28] Fondon J.W., Garner H.R. (2004). Molecular origins of rapid and continuous morphological evolution. Proc. Natl. Acad. Sci. USA.

[29] Hecht E.E., Smaers J.B., Dunn W.D., Kent M., Preuss T.M., Gutman D.A. (2019). Significant neuroanatomical variation among domestic dog breeds. J. Neurosci..

[30] Boyko A.R., Quignon P., Li L., Schoenebeck J.J., Degenhardt J.D., Lohmueller K.E., Zhao K., Brisbin A., Parker H.G., vonHoldt B.M. (2010). A simple genetic architecture underlies morphological variation in dogs. PLoS Biol..

[31] Finka L.R., Luna S.P., Mills D.S., Farnworth M.J. (2020). The application of geometric morphometrics to explore potential impacts of anthropocentric selection on animals’ ability to communicate via the face: The domestic cat as a case study. Front. Vet. Sci..

[32] Vilaseca C., Méndez M.A., Pinto C.F., Benítez H.A. (2020). Assessment of shape variation patterns in Triatoma infestans (Klug 1834) (Hemiptera: Reduviidae: Triatominae): A first report in populations from Bolivia. Insects.

[33] Galimberti F., Sanvito S., Vinesi M.C., Cardini A. (2019). “Nose-metrics” of wild southern elephant seal (Mirounga leonina) males using image analysis and geometric morphometrics. J. Zool. Syst. Evol. Res..

[34] Kirchengast S. (2019). Asymmetry patterns are associated with body size and somatic robustness among adult! Kung San and Kavango people. Anthropol. Rev..

